# The Heating and Lighting of Hospital Wards

**Published:** 1909-01-23

**Authors:** 


### HOSPITAL ADMINISTRATION.

### CONSTRUCTION AND ECONOMICS.

### THE HEATING AND LIGHTING OF HOSPITAL WARDS.

### II. HEATING BY RADIATORS.

As explained in the last article, the radiator is simply a nest of pipes arranged, in the latest forms, in ornamental fashion. The earliest form of radiator consisted of a plain iron pipe running round the skirting boards of the wards, and heating the air in contact with it, convection currents being set up in the usual way. For certain purposes, such as for heating a number of small adjoining rooms, the hot pipe answers very well; but for large rooms, such as wards, it is hardly satisfactory. The next arrangement was the forming of the pipes into grids, such as are used with steam, for all kinds of purposes, where heat is required to be taken from the steam; this has gradually led on to the form with which readers of The Hospital will be familiar, in which a number of tubes, made in a particular form, having a large surface, are fixed together upon a metal base, arranged so that the tubes stand a few inches above the floor, and also arranged that the air to be warmed can pass freely through them. Radiators made in this way may be used to deliver heat at any part of a ward by standing directly out in the ward between beds, or in the middle of the ward; or they may be used to heat air which is brought in from outside through holes in the wall protected by gratings. A very good system of heating can be arranged by using radiators in both ways.

The radiators themselves may be heated by the passage of hot water or steam through the tubes of which they are composed, and in a few cases they have been arranged to be heated by gas directly, and also indirectly, the tubes being filled with water, heated by Bunsen burners. In all cases the principle of working is the same. The fuel which furnishes the heat is consumed either in the furnace of a boiler in the basement, or, in the case of gas, at the gasworks, and the heat generated by the burning fuel is distributed either by hot water, steam, or gas. In the case of gas-heated radiators or gas-heated water radiators there is the secondary consumption of fuel in the place to be heated itself; but the consumption of coal and the arrangements for handling the coal are at the gasworks.

In the case of electric radiators the fuel is consumed where the motive power is employed to drive the dynamo machines, and this may be in the basement, or in an outbuilding of the hospital, or at the town or county electricity generating station.

The principle of the distribution of the heat is the same in all cases. With hot water or steam, pipes convey fluids in which heat is stored, from the place where the fuel has been consumed to the wards to be heated. In the case of gas and electricity, pipes or copper conductors carry gas or electricity from the point where both are generated to the place to be heated, where, by secondary combustion in the case of gas, and by direct conversion in the case of electricity, heat is produced exactly at the point where it is wanted.

There are two methods of hot-water heating, known as high and low pressure; and two methods of steam heating, also known as high and low pressure. The differences between these are those of temperature. With low-pressure hot water the water circulates at a temperature of 170° to 180° F., and usually loses about 10° to 20° in the process. With high-pressure hot water the water is heated to a temperature considerably above boiling-point, special arrangements being made to enable the water to be circulated at the high temperatures named, without being converted into steam. With high-pressure hot water, temperatures of 500° F. are sometimes reached in the circulating medium, and those of 300° to 350° are quite common. With high and low pressure steam the temperatures range from 230° up to 300° F. odd. There is, however, one important difference between the use of steam and the use of hot water. With hot water the water itself receives the heat and parts with it, through the medium of the radiator surfaces, to the air in their neighbourhood, returning to the heating apparatus, where its temperature is again raised and it again commences to circulate. With steam the water to be employed in heating is converted into steam, and in so doing absorbs a very much larger amount of heat than water is able to. Hence, higher temperatures are obtained by the use of steam with smaller radiator surfaces than are possible with merely heated water. On the other hand, heating water is more economical than converting it into steam, because the latent heat absorbed in converting the water into steam is rarely recovered in useful heat in the wards. In those hospitals, however, where steam is employed for driving engines of any kind, as for dynamo machines for lighting or any other purpose, the exhaust steam can be employed either to heat radiators directly, or to heat water by the aid of apparatus known as calorifiers, to any temperature that may be desired.

Electrical radiators are of two principal forms—those in which long incandescent electric lamps are employed, and those in which specially prepared conductors are used, the conductors being enclosed in cases designed to give off heat and to set up convection currents in a similar manner to the hot-water and steam radiators. In the latest form of electric radiator, in fact, the hot-water radiator has been closely copied. With the incandescent-lamp radiators, lamps nine inches long are usually employed, fixed two, three, or four in an ornamental fitting, with a reflector behind.

In all cases the radiators should be arranged so that those in the room to be warmed can control the heat given off. With steam, hot water and gas the rate of delivery is controlled by a valve.

